# Development and characterization of a double-crested cormorant hepatic cell line, DCH22, for chemical screening

**DOI:** 10.3389/ftox.2025.1482865

**Published:** 2025-02-12

**Authors:** Tasnia Sharin, Doug Crump, Jason M. O’Brien

**Affiliations:** National Wildlife Research Centre, Environment and Climate Change Canada, Ottawa, ON, Canada

**Keywords:** DCH22 cell line, spheroid, hepatocyte, metabolism, vitellogenin, *CYP1A*, *CYP3A*

## Abstract

There are currently no available cell lines for the ecologically relevant colonial waterbird species, the double-crested cormorant (DCCO). DCCOs are high trophic level aquatic birds that are used for routine contaminant monitoring programs in the Laurentian Great Lakes and marine coasts of Canada. Developing a DCCO cell line for *in vitro* toxicological screening will ideally provide improved understanding of the effects of environmental chemicals given the large differences in sensitivity between laboratory and wild avian species. In this study, an immortalized DCCO hepatic cell line, DCH22, was established from the liver of a day 22 female embryo as a potential alternative to primary DCCO embryonic hepatocytes (DCEH) for chemical screening. DCH22 cells were cultured for over a year and have hepatocyte-like morphology. Exposure to 3,3′,4,4′,5-pentachlorobiphenyl (PCB-126), benzo-a-pyrene, ß-napthoflavone and phenacetin induced *CYP1A* activity and mRNA expression in DCH22 3D spheroids. Induction of *CYP3A* activity and mRNA expression was observed following exposure to hexabromocyclododecane (HBCD), tris(1,3-dichloroisopropyl)phosphate, carbamazepine, and metyrapone. The phase II metabolism gene, UGT1A1, was upregulated following HBCD exposure and DCH22 spheroids expressed vitellogenin protein after exposure to 17α-ethinylestradiol. Based on these data, the novel DCH22 cell line, cultured as 3D spheroids, has potential use as an alternative to DCEH for chemical screening and will permit the evaluation of avian species differences in sensitivity from an *in vitro* screening perspective.

## 1 Introduction

There is an international demand for faster, focused, ethical, and economical toxicity testing tools, and a move towards mechanistic toxicology. High-throughput *in vitro* approaches can help meet the demands of this shifting toxicity testing paradigm (Cacciamali et al., 2022). Primary embryonic hepatocyte culture is a commonly used *in vitro* model for avian toxicity testing (Pagé-Larivière et al., 2018; Head and Kennedy, 2010). However, it is difficult to obtain eggs of avian wildlife species for the preparation of primary embryonic hepatocytes and such studies can only be conducted during the breeding season. Furthermore, primary hepatocytes have limited proliferation and metabolic activity, they can only be maintained for a few days, and they require the use of animals (Soldatow et al., 2013). Thus, it is a challenge to screen large numbers of chemicals using primary hepatocyte culture. Immortalized cell lines can be cultured indefinitely, permit the generation of large amounts of mechanistic and dose-response data, and reduce/replace the use of animals (Astashkina and Grainger, 2014). Advancements in cell culturing techniques, such as three-dimensional (3D) culture systems, have made immortalized cell lines more relevant for toxicity testing because cells cultured as 3D spheroids are more predictive of *in vivo* responses to chemicals compared to immortalized cells cultured as 2D monolayers (Cacciamali et al., 2022).

There are two commercially available avian hepatic cell lines, leghorn male hepatoma (LMH) and LMH/2A, both derived from chicken (*Gallus gallus domesticus*), a key laboratory model for avian toxicity testing. Conversely, there are no hepatic cell lines available for ecologically relevant avian species, such as the double-crested cormorant (*Nannopterum auritum*; DCCO). DCCOs are piscivorous colonial waterbirds that occupy a high trophic position in the aquatic food web and therefore, are used as wildlife indicator species in freshwater (e.g., Great Lakes) and marine environments across Canada (Harris et al., 2005). Primary DCCO embryonic hepatocytes (DCEH) have been used to screen chemicals and compare toxicity of chemicals between laboratory and wildlife species; e.g., chicken versus DCCO (Pagé-Larivière et al., 2018; Crump et al., 2016). A cell line for an ecologically relevant avian species may represent a better predictor of the toxic effects of environmental chemicals in wildlife, since previous studies have found that chickens are more sensitive to a range of chemicals than wild species (Nordén et al., 2016; Head et al., 2008). For example, chickens are more sensitive to arylhydrocarbon receptor (AhR) agonists, such as dioxin-like compounds, which induce *CYP1A* activity and other AhR related genes and proteins (Karchner et al., 2006). The objectives of this study were to develop a continuous hepatic cell line from embryonic DCCO and to evaluate its utility as an animal-free alternative to DCEH for toxicity testing.

## 2 Materials and methods

### 2.1 Hepatocyte isolation and cell line development

All procedures involving the handling of animals were conducted according to protocols approved by Environment and Climate Change Canada’s Wildlife Eastern Animal Care Committee. Fertilized, unincubated DCCO eggs were collected from ground nests containing one or two eggs from a nesting colony in Batchawana Bay, Lake Superior (46°52′8.89″N; 84°26′49.45″W) that has low concentrations of legacy persistent organic pollutants and chemicals of emerging concern in avian eggs (de Solla et al., 2016). DCCO eggs were transported in foam-lined coolers to the National Wildlife Research Centre in Ottawa and incubated according to methods described previously (Powell et al., 1997; Crump et al., 2010) within 48 h of collection. Briefly, DCCO eggs were placed horizontally in a Petersime incubator (Model XI) at 37.5°C and 60% relative humidity. An embryonic day 22 female DCCO was euthanized by decapitation (sex was confirmed via gonadal identification during dissection) and the liver was dissected and rinsed in HEPES buffer. Subsequently, the liver was placed in a petri dish with 10 mL collagenase solution, sliced into small pieces, transferred to a tube and digested at 37°C for 5 min. The resulting solution was centrifuged at 500 g for 5 min, the supernatant and red blood cells were removed, and cells were suspended in 5 mL collagenase solution and centrifuged again at 250 g for 4 min. After the collagenase solution was aspirated, 10 mL of 2% bovine serum albumin (BSA) solution was added to the pellet and centrifuged at 250 g for 4 min. The resulting pellet weighed 0.43 g and was suspended in hepatocyte growth media, which consisted of DMEM/F12 solution supplemented with 10% fetal bovine serum (FBS), 1% penicillin/streptomycin, 20 ng/mL mouse hepatocyte growth factor (HGF), 10 ng/mL mouse oncostatin M (OSM), 0.1% DMSO, 0.5 μg/mL insulin, 0.01 µM hydrocortisone, 0.025 µM dexamethasone and 10 mM nicotinamide. All reagents were purchased from Sigma-Aldrich (MO, United States), except HGF and OSM, which were purchased from R&D Systems (MN, United States). Hepatocytes (viability ∼70%, stained with trypan blue, Countess automated cell counter (ThermoFisher Scientific, MN, United States)) were plated at a high density–eight million cells in a 100 mm petri dish (Nunc) coated with 0.2% gelatin and cultured at 37°C with 5% CO_2_. Media was changed every 2–3 days depending on the pH (less than 7) of the media. Cells were passaged (1:2 or 1:3 dilution) when they reached confluency by adding 3 mL trypsin-EDTA to the dish for 3 min, followed by inactivation of trypsin by adding 3 mL of media and centrifuging at 250 g for 3 min. The resulting pellet was resuspended in 2 or 3 mL of the media depending on the size of the pellet and aliquoted to flasks containing 10 mL media. From passage 5, cells had hepatocyte-like morphology and formed small clusters. After 6 passages (∼1.5 months in culture), the culture media was modified to hepatocyte maturation media. This media consisted of DMEM/F12 media supplemented with 10% FBS, 1% penicillin/streptomycin, 0.5% DMSO, 1% insulin transferrin selenium (ITS) solution, 20 ng/mL HGF, 10 ng/mL mouse epidermal growth factor (EGF), 0.1 µM hydrocortisone, 10 nM dexamethasone and 5 mM nicotinamide. The media was changed every 48 h and cells were passaged when they reached 80%–90% confluency. After 20 passages (∼11 months), the culture media was modified to maintenance media, which consisted of DMEM/F12 supplemented with 10% FBS, 1% penicillin/streptomycin, 0.1% DMSO, 1% ITS and 1 µM hydrocortisone. Cells were frozen in 5% DMSO in a −80°C ultra-low freezer or the vapour phase of liquid nitrogen starting from passage 5. Upon thawing, cells were centrifuged at 300 g for 3 min and the resulting pellet was resuspended in maintenance media and cultured occasionally to check for proliferation. The cells were renumbered as passage 0 (P0) after thawing the frozen stock. Cells were checked for *mycoplasma* contamination using 4′,6-diamidino-2-phenylindole (DAPI) staining, and no contamination was detected.

For chemical exposure experiments, maintenance media, with all reagents mentioned above with the exception of 0.1% DMSO, was used to culture cells. Cells were revived from liquid nitrogen vapour phase and cells from passage 6–8 were used for the DCH22 characterization experiments. For 3D spheroids, 20,000 cells in 250 μL of medium were added to each well of a 96 well ultra-low attachment (ULA) microplate (Corning, NY, United States). Medium was changed daily by removing 200 μL existing medium and adding 200 μL fresh medium to each well. Spheroids were cultured for 4 days prior to chemical administration at 37°C with 5% CO_2_. All chemicals were prepared in DMSO. The optimal dosing concentrations were determined by measuring cell viability using the CellTiter-Glo 3D Cell Viability Assay (Promega, WI, United States) to enable differentiation of cytotoxic versus assay-specific responses.

### 2.2 Phase I and II gene expression

DCH22 spheroids were dosed with DMSO, 3,3′,4,4′,5-pentachlorobiphenyl (PCB-126; Cas # 57465-28-8, 99% purity, 0.1–100 nM) and benzo-a-pyrene (BaP; Cas # 50-320-8, 96% purity, 0.1–100 µM) for CYP1A4 (phase I), and hexabromocyclododecane (HBCD; Cas # 3194-55-6, 99% purity, 0.1–30 µM) for CYP3A37 (phase I) and UGT1A1 (phase II) gene expression. RNA was extracted from spheroids (n = 3/treatment) using a RNeasy kit (Qiagen, MD, United States) following the manufacturer’s instruction. RNA concentration (30–33 ng/μL) and purity (A260/280 nm = 1.9–2.1) were determined using a QIAxpert (Qiagen). 30 ng of RNA was reverse transcribed to cDNA using a QuantiTect reverse transcription kit (Qiagen) according to the manufacturer’s protocol. CYP1A4*,* CYP3A37*,* UGT1A1, and RPL4 primers (Supplementary Table S1) were obtained from IDT technologies (IA, United States). cDNA samples were diluted 1:10 with RNase free water before PCR. Real-time PCR reactions were prepared with RT2 SYBR Green Rox mastermix following the manufacturer’s protocol (Qiagen). The thermal profile was 95°C for 10 min followed by 40 cycles of 95°C for 15 s and 60°C for 1 min and final extension at 95°C for 1 min. Real-time PCR was performed using a Stratagene Mx3005 (Agilent Technologies, CA, United States). Data were analysed using the 2^−ΔCt^ method (Schmittgen and Livak, 2008) and normalized to the reference gene, RPL4. Fold change was determined relative to the respective DMSO control group. Significant differences from DMSO control were determined by one-way ANOVA followed by Bonferroni’s multiple comparisons test using GraphPad Prism Ver. 6.07.

### 2.3 CYP1A (EROD) activity

For the 7-ethoxy-resorufin-O-deethylase (EROD) assay, DCH22 3D spheroids were dosed (n = 3) with 1.25 µL of the dimethyl sulfoxide (DMSO (0.5%); Sigma-Aldrich) vehicle control or 0.01–100 nM PCB-126, 0.1–100 µM BaP, 0.1–100 µM ß-napthoflavone (Cas # 6051-87-2; 98% purity), or 0.1–100 µM phenacetin (Cas # 62-44-2, 98% purity). All test chemicals were dissolved in DMSO to attain in-well, nominal concentrations (listed above) in 250 µL of medium per well. The cells were incubated for 24 h, media aspirated, and plates were flash frozen on dry ice and stored at −80°C. EROD activity was determined as described previously (Sharin et al., 2020). The presence of the EROD product, resorufin, was measured at an excitation wavelength of 530 nm and an emission wavelength of 590 nm and total protein concentration was measured at an excitation wavelength of 400 nm and emission wavelength of 460 nm using a DTX 880 multimode microplate reader (Beckman Coulter, CA, United States). Replicate wells were analyzed and EROD activity was reported as pmol resorufin/min/mg protein. Significant differences in activity between treatment and control were determined with one-way ANOVA (p < 0.05), followed by Dunnett’s multiple comparison test using GraphPad Prism Ver. 6.07.

### 2.4 CYP3A activity

For the *CYP3A* assay, DCH22 spheroids were dosed (n = 5) with 1.25 µL of the DMSO (0.1%) vehicle control or 1–30 µM HBCD, 0.1–100 µM tris(1,3-dichloroisopropyl)phosphate (TDCPP, Cas # 13674-87-8, >90% purity), 0.1–500 µM carbamazepine (Cas # 298-46-4, 98% purity) or 0.1–500 µM 2-methyl-1,2-di-3-pyridyl-1-propanone (metyrapone; Cas # 54-36-4, 98% purity), dissolved in DMSO to yield in-well nominal concentrations in 250 µL of medium per well. *CYP3A* activity was measured using the P450-Glo *CYP3A4* Assay and Screening System (Promega, WI, United States) following the manufacturer’s instructions. Briefly, 12.5 µL of 4X *CYP3A4* reaction mixture was added to each well, mixed for 1 min on a plate shaker, and incubated for 10 min at 37°C. Next, 25 μL of 2X NADPH regeneration system was added to all wells and incubated for an additional 10 min at 37°C. 50 μL luciferin detection reagent was added to all wells, mixed for 1 min on a plate shaker and incubated for 20 min at 37°C. Luminescence was measured using a Luminoskan Ascent luminometer (ThermoFisher Scientific). *CYP3A* luminescence was determined by subtracting the luminescence from minus-P450 control (glo substrate, buffer, and NADPH regeneration system). Five technical replicate wells per dose group were analyzed and *CYP3A* activity was reported as relative luminescence units (RLU). Significant differences in activity between treatment and control were determined with one-way ANOVA (p < 0.05), followed by Dunnett’s multiple comparisons test using GraphPad Prism Ver. 6.07.

### 2.5 Vitellogenin concentration

DCH22 spheroids were dosed with DMSO or 0.1–100 µM 17α-ethinylestradiol (EE2, Cas# 57-63-6, 98% purity) in phenol red-free DMEM/F12 media supplemented with 10% FBS, 1% penicillin/streptomycin, 1% insulin transferrin selenium (ITS) solution and 1 µM hydrocortisone. Phenol red-free media was used to avoid interference of phenol red with fluorescence readings and because it can act as a weak estrogen in some cell types and lines (Berthois et al., 1986). Incubation time was 48 h as opposed to the assays described above for EROD, *CYP3A* activity and gene expression (24 h). Vitellogenin (*VTG*) concentrations in spheroid lysate and culture medium (n = 3/dose) were determined using a chicken *VTG* ELISA Kit (Abbexa Ltd., Cambridge, United Kingdom) following the manufacturer’s protocol. Optical density at 450 nm was determined using a DTX880 multimode microplate reader (Beckman Coulter). *VTG* concentration was determined from the standard curve. Significant differences in concentration between treatment and control were determined with one-way ANOVA (p < 0.05), followed by Dunnett’s multiple comparisons test using GraphPad Prism Ver. 6.07.

## 3 Results and discussion

There is a lack of toxicity data for ecologically relevant avian indicator species such as DCCOs, which are naturally exposed to chemicals in the environment. DCCOs have been used as a model wildlife species to monitor the prevalence and effects of contaminants in aquatic ecosystems (Harris et al., 2005). The liver is the primary site of xenobiotic metabolism and biotransformation and is an important organ for toxicity testing (Soldatow et al., 2013). Currently, there are no immortalized hepatic cell lines available for DCCO and thus, when conducting *in vitro* screening approaches for this species, primary hepatocytes must be used. Primary hepatocyte preparation is limited by the need to collect freshly laid eggs from breeding sites (ideally that have low levels of contaminants) and the time of year (breeding season only). The goal of this study was to derive a DCCO hepatic cell line, DCH22, and characterize metabolic activity and response to estrogenic compounds to evaluate whether it could be used as an alternative to primary DCEH for chemical screening.

**FIGURE 1:**
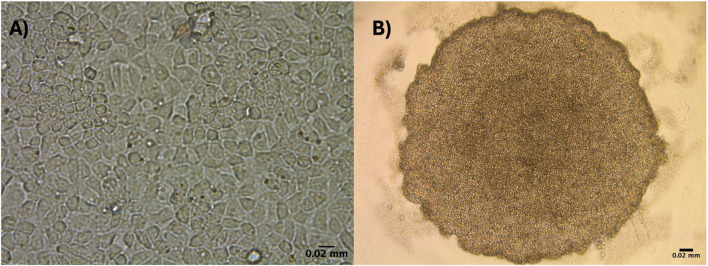
DCH22 cells in two culture conditions grown in 96-well plates: **(A)** 2D monolayer confluent cells, and **(B)** 3D spheroid. Scale bar = 0.02 mm.

DCH22 cells were derived from the liver of an embryonic day 22 female using growth factors (EGF, HGF, OSM) and small molecules including, dexamethasone and nicotinamide. These factors aid in hepatocyte growth, maturation and maintenance of hepatic functions (Dong et al., 2008). DCH22 cells were continuously cultured for over a year and thus, can be considered a cell line. The cells were cryopreserved in the vapour phase of liquid nitrogen and upon thawing, retained metabolic activity (*CYP1A* and *CYP3A*). The doubling time of the cells is between 23 and 26 h. After the initial five passages, the morphology of 2D confluent monolayer cells was predominantly flat polygonal cells that grew in clusters (Figure 1A; Supplementary Figure S1). DCH22 cells self-assembled and formed spheroids by 24 h without any exogenous extracellular matrix when cultured in ULA 96 well microplates (Figure 1B; Supplementary Figure S1). For DCH22 cell line characterization, the cells were cultured as spheroids because spheroids have been shown to have improved cell differentiation, enzyme activity and chemical metabolism (Sharin et al., 2021; Cacciamali et al., 2022).

### 3.1 Phase I and II mRNA expression in DCH22 3D spheroids

**FIGURE 2:**
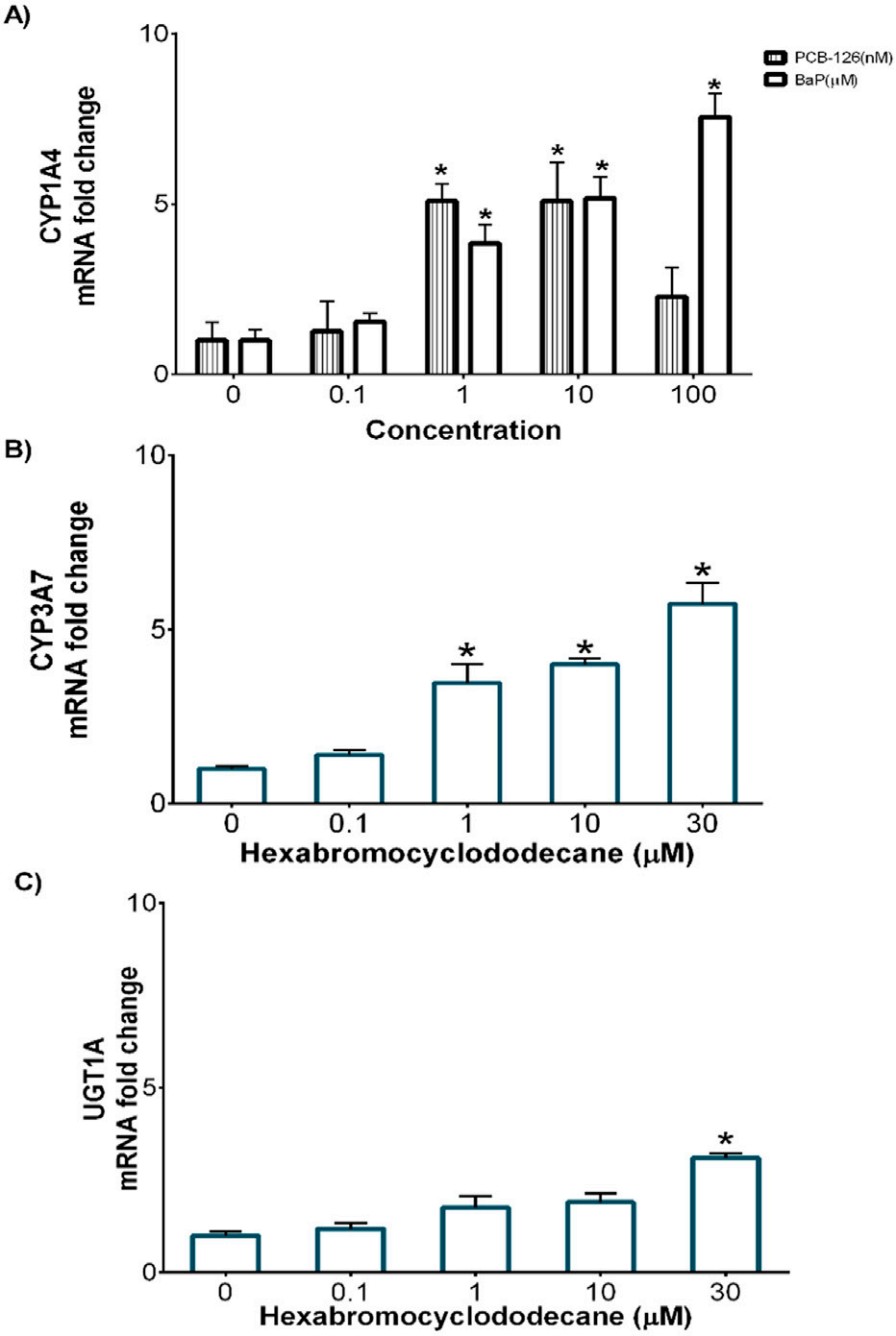
Concentration-dependent fold change of mRNA expression for **(A)** Cyp1a4 following exposure to BaP and PCB-126, **(B)** Cyp3a37 and **(C)** Ugt1a1 following HBCD exposure in DCH22 spheroids after 24 h. “*” denotes significant differences compared to untreated spheroids (p ≤ 0.05).

DCH22 spheroids expressed Phase I and II metabolism genes (CYP1A4, CYP3A7 and UGT1A1), which are effective markers of hepatocytes and metabolic competency. Basal expression (Ct values) of CYP1A4, CYP3A7, UGT1A1 and the housekeeping gene, RPL4, were 34, 35, 28 and 25, respectively. Exposure to PCB-126, BaP and HBCD resulted in a concentration-dependant increase in CYP1A4 (1.5 to 7.6-fold) and CYP3A37 (1.5 to 5.7-fold) mRNA expression, respectively (Figures 2A, B). The decrease observed in CYP1A4 expression at 100 nM PCB-126 was due to cytotoxicity. Upregulation of CYP1A4 gene expression by PCB-126 and BaP have been observed in different species, including birds (Eng et al., 2017; Santos et al., 2018). Maximal induction of CYP1A4 mRNA expression after 10 nM PCB-126 and 10 µM BaP exposure in DCH22 was lower than LMH spheroids, where exposure to these compounds resulted in 49-fold and 52-fold increases in expression at similar concentrations (Sharin et al., 2020; Sharin et al., 2021). Expression of UGT1A1 was upregulated by HBCD at the highest concentration in DCH22 (3.1-fold) (Figure 2C). In previous studies, HBCD exposure upregulated CYP3A37 in chicken primary hepatocytes (10-fold at 10 µM HBCD) and UGT1A1 expression in embryos (5-fold at 1,000 ng/g egg) (Crump et al., 2010; Crump et al., 2008).

### 3.2 CYP1A activity in 3D spheroids

**FIGURE 3:**
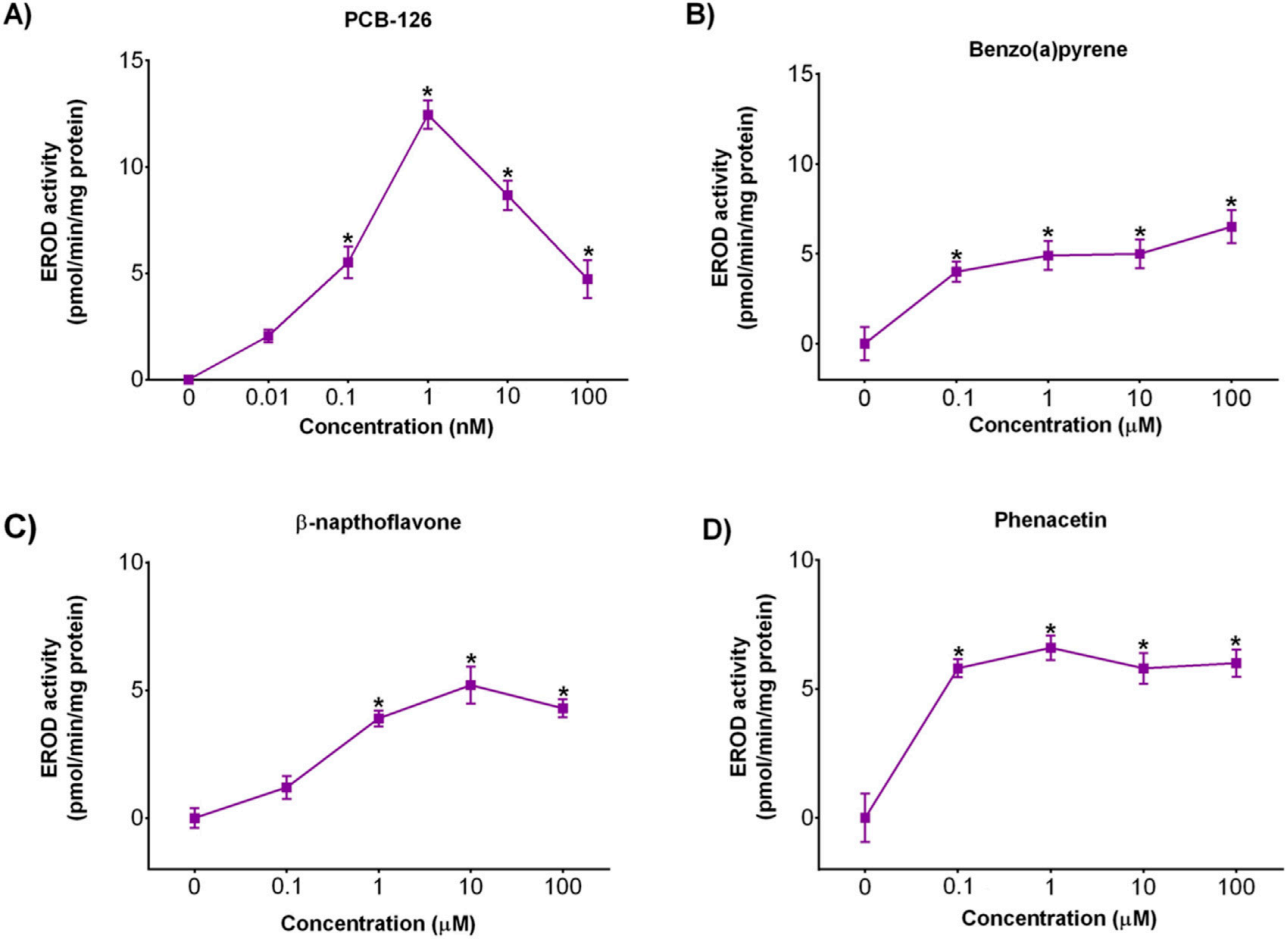
Concentration-dependent effects of four CYP1A inducers **(A)** PCB-126, **(B)** BaP, **(C)** ß-napthoflavone, and **(D)** phenacetin on EROD activity in DCH22 3D spheroids following 24 h exposure. Data are presented as mean and error bars represent SEM, p ≤ 0.05. “*” denotes significant differences compared to untreated spheroids.

Induction of EROD activity in birds is a widely used and accepted biomarker of exposure to chemicals that induce *CYP1A* activity via AhR binding (Farmahin et al., 2016). Exposure to PCB-126, BaP, ß-napthoflavone and phenacetin for 24 h led to an increase in *CYP1A* induction/EROD activity in DCH22 3D spheroids (Figure 3). PCB-126 is a dioxin-like compound, which binds to the AhR and upregulates the expression of *CYP1A* (Crump et al., 2016). The decrease in *CYP1A* activity observed at higher concentrations of PCB-126 is due to cytotoxicity and likely competitive inhibition of *CYP1A* inducers (Petrulis and Bunce, 1999). The induction of *CYP1A* activity in DCH22 spheroids (max induction = 12.6 pmol/min/mg protein) followed a similar pattern observed in DCEH, but the activity was much higher in DCEH (∼200 pmol/min/mg protein) (Crump et al., 2016). A similar finding of variable maximal activity was observed when comparing EROD activity between chicken embryonic hepatocytes and LMH spheroids following exposure to PCB-126, where the pattern was similar (including EC50 values), but the activity was greater in CEH (Sharin et al., 2020). BaP is another AhR agonist, which is metabolically activated by *CYP1A* and induces *CYP1A* activity in chicken LMH cells cultured as spheroids (8.54 pmol/min/mg protein) (Sharin et al., 2021). The increase in *CYP1A* activity at higher concentrations of BaP could be due to BaP metabolism to BP-7,8-diol, which is further metabolized to BPDE via *CYP1A* activity (Shiizaki et al., 2017). ß-napthoflavone is a well-known AhR agonist and was found to increase *CYP1A* activity in rat liver slices (<50 pmol/min/mg protein), zebrafish embryos, and the human hepatic HepG2/C3A cell line (Glöckner et al., 1998; Boehler et al., 2018; Liu et al., 2015). A similar ß-napthoflavone *CYP1A* activity curve (i.e., decrease at high concentration) was observed in rainbow trout and European eel (Fenet et al., 1998). Finally, the non-steroidal anti-inflammatory drug, phenacetin, is another well-characterized inducer of *CYP1A* activity in humans and rodents (Warrington et al., 2004). Phenacetin is metabolized by *CYP1A* to acetaminophen, which is also metabolized by *CYP1A*, which could account for the sustained *CYP1A* activity at high concentrations (Hinson, 1983). There are no data on the activation of *CYP1A* by ß-napthoflavone or phenacetin in birds prior to this work. Overall, *CYP1A* activity in DCH22 spheroids was similar to other cell lines, but much lower than primary hepatocyte culture or liver slices.

### 3.3 CYP3A activity in 3D spheroids

**FIGURE 4:**
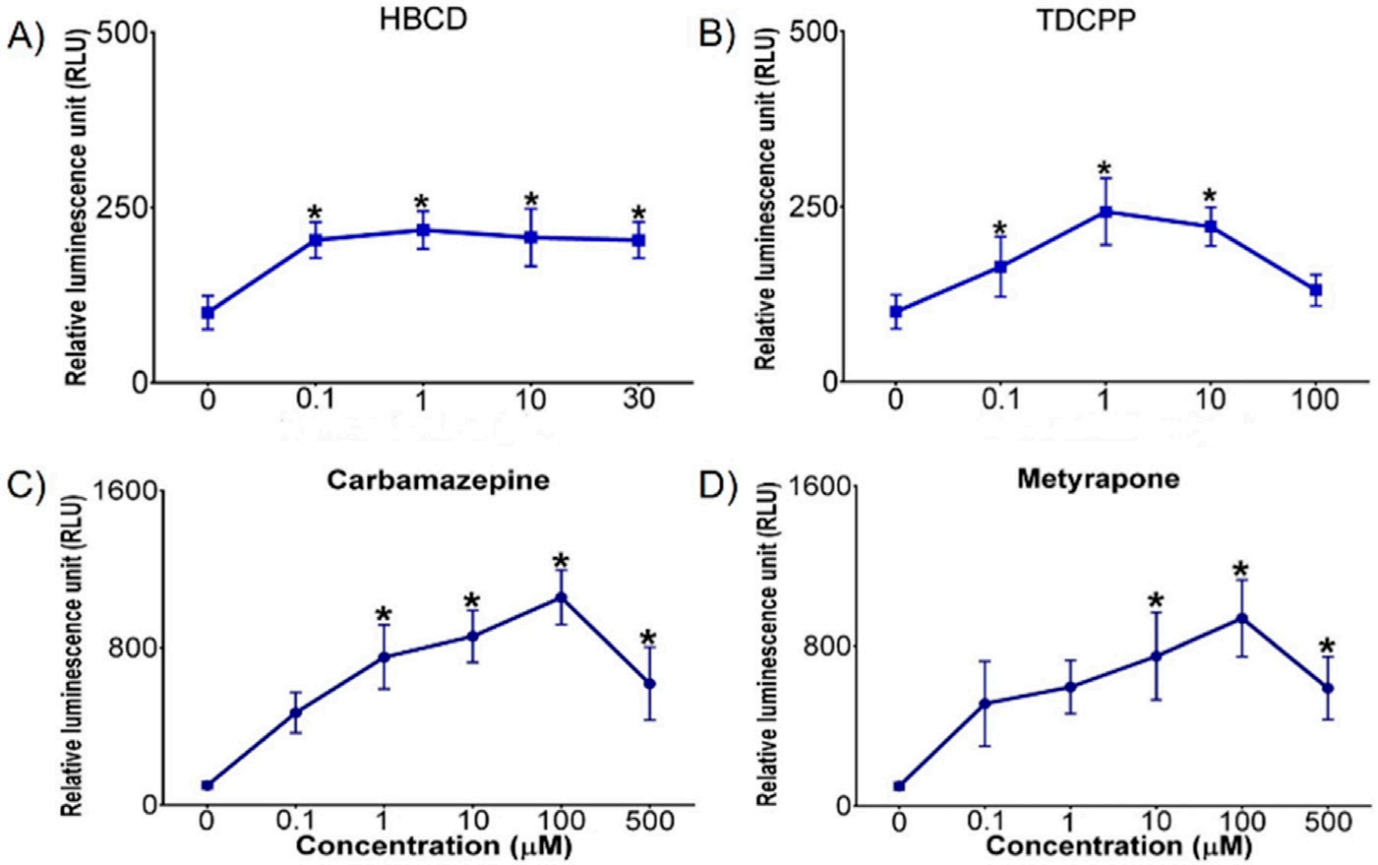
Concentration-dependent effects of **(A)** HBCD, **(B)** TDCPP, **(C)** carbamazepine, and **(D)** metyrapone on CYP3A activity (measured as relative luminescence units [RLU]) in DCH22 3D spheroids after 24 h exposure. Data are presented as mean and error bars represent SEM, p ≤ 0.05. “*” denotes significant differences compared to untreated spheroids.

*CYP3A* metabolizes a vast number of chemicals with various chemical structures and is the predominant CYP450 in the mammalian liver (Ourlin et al., 2000). Chicken xenobiotic receptor (CXR) has high homology with mammalian pregnane X receptor (PXR) and constitutive androstane receptor (CAR) and mediates *CYP2* and *CYP3* activity (Handschin et al., 2000). *CYP3A* activity increased in DCH22 3D spheroids upon exposure to HBCD, TDCPP, carbamazepine and metyrapone (Figure 4). HBCD is a brominated flame retardant and induces *CYP3A* activity in the liver via PXR in mammals (Proença et al., 2023). *CYP3A* activity increased after exposure to 0.1 µM HBCD and remained constant at higher doses. TDCPP is a chlorinated organophosphate flame retardant, which activates PXR and induces *CYP3A* activity in mice (Tenlep et al., 2023). HBCD (10 µM) and TDCPP (10 µM) upregulated CYP3A37 mRNA expression 8- and 10-fold in primary chicken embryonic hepatocytes, respectively, and in chicken embryos (15-fold at 1,000 ng/g egg HBCD and 62-fold at 50 μg/g egg TDCPP, respectively) (Crump et al., 2008; Farhat et al., 2014). Carbamazepine, an anticonvulsant medication, is metabolized by *CYP3A* to its active metabolite in the liver and activates the CAR pathway in humans (Bolleddula et al., 2022). Metyrapone, a glucocorticoid synthesis inhibitor, is an inducer of *CYP3A* expression and activates PXR in human and rat hepatocytes (Harvey et al., 2000; Wright et al., 1996). The decrease in *CYP3A* activity at the highest dose of TDCPP, carbamazepine and metyrapone is likely due to competitive inhibition (as observed for certain *CYP1A* agonists described above in *CYP1A* activity in 3D spheroids). There is no available information on the induction of *CYP3A* by HBCD, TDCPP, carbamazepine or metyrapone in DCEH and thus, a comparison to primary hepatocytes of this species was not possible. In a previous study, metyrapone upregulated mRNA expression of CYP3A37 (80-fold at 1 mM) in the immortalized chicken hepatic cell line, LMH (Ourlin et al., 2000).

### 3.4 Vitellogenin concentration in DCH22 3D spheroids

**FIGURE 5:**
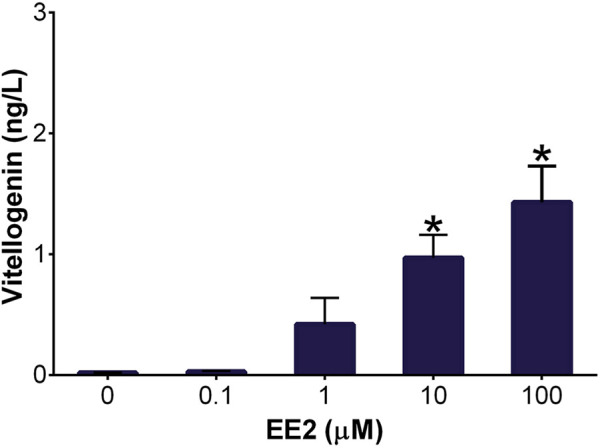
Vitellogenin concentration in DCH22 spheroids and culture medium after exposure to EE2 for 48 h. Data are presented as mean and error bars represent SEM. “*” denotes significant differences compared to untreated spheroids (p ≤ 0.05).

The presence of vitellogenin was used as a hepatocyte marker for the DCH22 cell line characterization. Vitellogenin is an egg yolk protein precursor synthesized in hepatocytes. It is not expressed in embryonic birds but exposure to estrogenic compounds can cause vitellogenin synthesis during this life stage. Thus, it is used as a biomarker of xenoestrogen exposure and is a useful endpoint for toxicity testing (Mullinix et al., 1976). Exposure to the synthetic estrogen, 17*α*-ethinylestradiol (EE2; 10 and 100 µM), for 48 h resulted in a concentration-dependant increase in vitellogenin concentrations (1 and 1.4 ng/L) in DCH22 spheroid lysate and culture medium (Figure 5). EE2 has been used as a positive control for vitellogenin induction in fish plasma and hepatocyte culture (Navas and Helmut, 2006; Fenske et al., 2001).

## 4 Conclusion

In this study, a continuous embryonic DCCO hepatic cell line, DCH22, was developed and characterized. The increase in *CYP1A* and *CYP3A* activity and mRNA expression in 3D spheroids following exposure to well-known environmental and pharmaceutical inducers indicates that the DCH22 cell line has phase I metabolic activity. Therefore, the DCH22 cell line is a suitable *in vitro* model to investigate the induction of phase I enzymes by diverse chemicals. Upregulation of UGT1A1 mRNA expression and vitellogenin protein further corroborate the utility of this cell line for chemical screening and confirm the hepatocyte nature of the cells based on the presence of these well-known markers. The DCH22 cell line has metabolic competence and can be used as an alternative to DCEH for toxicity testing. Comparisons to *in vitro* studies with other hepatic avian cell lines will permit the evaluation of species differences in sensitivity to chemicals for avian toxicity testing without requiring the field collection of eggs.

## Data Availability

The datasets presented in this study can be found in online repositories. The names of the repository/repositories and accession number(s) can be found in the article/supplementary material.
