# Supplementary material for: Development and characterization of a double-crested cormorant hepatic cell line, DCH22, for chemical screening
Source: Front Toxicol. 2025 Feb 12;7:1482865. doi: 10.3389/ftox.2025.1482865 (PMC11861107; doi:10.3389/ftox.2025.1482865)
Supplement: Supplementary file 1 [file Table1.docx]

**Supplementary Information**

**Table S1.** Primer sequences used to amplify the gene targets

| **Gene** | **Forward Sequence (5’-3’)** | **Reverse Sequence (5’-3’)** |
| --- | --- | --- |
| CYP1A4 | CTTGTCCTGGAGCCTCATGT | CCTCTCATGGCGTATGGTCT |
| CYP3A37 | CTTCTTCCCCAGAGATGCCG | GTATCTCGATGTCGCTCAGGG |
| UGT1A1 | TATGCAATGCGGTGCCAATG | GGTCTGTCGAGGTGAAGGTC |
| RPL4 | AGAAGCGTTACGCCATCTGT | CGCTGGGAGGCATAAACCTT |
