# Supplementary material for: Development and characterization of a double-crested cormorant hepatic cell line, DCH22, for chemical screening
Source: Front Toxicol. 2025 Feb 12;7:1482865. doi: 10.3389/ftox.2025.1482865 (PMC11861107; doi:10.3389/ftox.2025.1482865)
Supplement: Supplementary file 2 [file DataSheet1.docx]

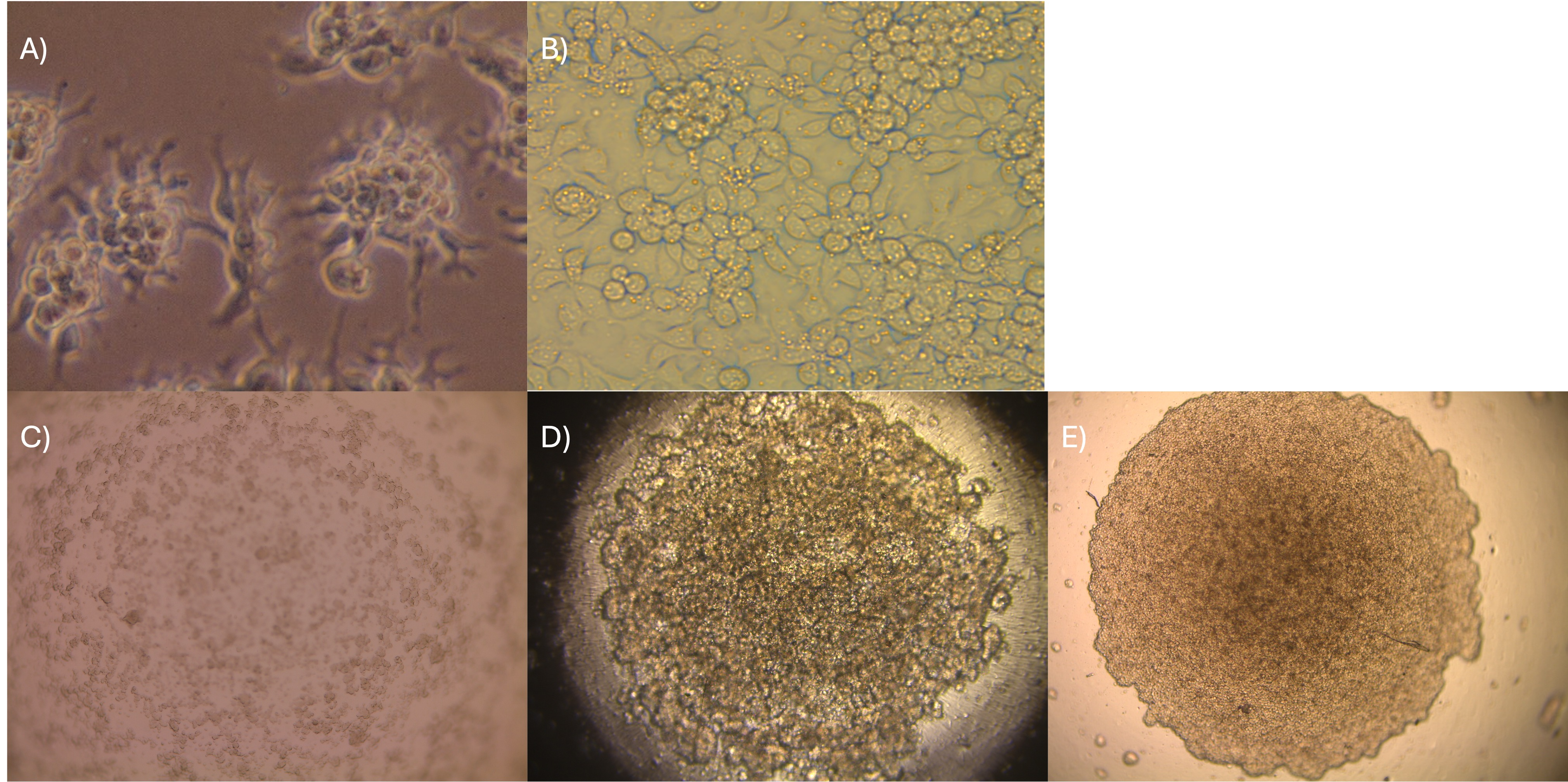


Figure S1. DCH22 cells after: A) 4 months culture, B) 2D monolayer culture, and 3D spheroid on C) Day 0, D) Day 1 and E) Day 4.
